# Development, quality, and influencing factors of colonoscopy in China: results from the national census in 2013 and 2020

**DOI:** 10.3389/fonc.2023.1276520

**Published:** 2023-09-22

**Authors:** Yun-Fei Jiao, Zhi-Yuan Cheng, Ye Gao, Chu-Ting Yu, Hui-Shan Jiang, Ting-Lu Wang, Ying Deng, Han Lin, Tian-Jiao Wang, Wei Wang, Rong Wan, Zhao-Shen Li, Lei Xin, Luo-Wei Wang

**Affiliations:** ^1^ Department of Gastroenterology, Changhai Hospital, Naval Medical University, Shanghai, China; ^2^ National Digestive Endoscopy Improvement System, Shanghai, China; ^3^ Department of Gastroenterology, Shanghai General Hospital, Shanghai Jiao Tong University School of Medicine, Shanghai, China

**Keywords:** colonoscopy, colorectal cancer, adenoma detection rate, cecal intubation rate, quality improvement

## Abstract

**Background and Aim:**

With the increasing burden of colorectal cancer (CRC), the practice of colonoscopy is gaining attention worldwide. However, it exhibits distinct trends between developing and developed countries. This study aims to explore its development and identify influencing factors in China.

**Methods:**

The Chinese Digestive Endoscopy Censuses were conducted twice in mainland China under the supervision of health authorities. Information regarding the practice of colonoscopy was collected through a structured online questionnaire. The authenticity of the data was evaluated through logical tests, and a random selection of endoscopic reports underwent manual validation by Quality Control Centers. Potential factors associated with colonoscopy were analyzed using real-world information.

**Results:**

From 2012 to 2019, the number of hospitals that performed colonoscopy increased from 3,210 to 6,325 (1.97-fold), and the volume increased from 5.83 to 12.92 million (2.21-fold). The utilization rate rose from 436.0 to 914.8 per 100,000 inhabitants (2.10-fold). However, there was an exacerbation of regional inequality in the adequacy of colonoscopy. Regions with higher incidence of CRC, higher gross domestic product per capita, more average numbers of endoscopists and tertiary hospitals tended to provide more accessible colonoscopy (P<0.001). Nationwide, the cecal intubation rate improved from 83.9% to 94.4% and the unadjusted adenoma detection rate (ADR) improved from 16.3% to 18.1%. Overall, hospital grading, educational background of endoscopists, economic income, and colonoscopy volume were observed as the significantly positive factors affecting ADR (P<0.05), but not the incidence of CRC or the number of endoscopists.

**Conclusions:**

Tremendous progress in colonoscopy has been made in China, but some issues needed timely reflection. Our findings provide timely evidence for better colonoscopy strategies and measures, such as quality control and medical education of endoscopists.

## Introduction

Over the past decades, colorectal cancer (CRC) has emerged as a significant public health concern, with its incidence ranking third globally among all cancer types, and second in terms of mortality in 2020 (1–3). Highly developed countries have witnessed stabilizing or decreasing CRC patterns and trends partly because of the screening effect and high-quality colonoscopy (3). However, CRC incidences are still rising dramatically in many low- and middle-income countries due to changes in lifestyle and diet (4). For instance, the burden of CRC continued to increase in China, the largest developing country with one-fifth of the world population. Currently, the crude incidence and mortality of CRC in China are 38.4 and 19.8 per 100,000, respectively (5), which means nearly 600,000 new cases and 310,000 deaths caused by CRC in just a single year (6).

However, the contradiction between increasing demand and the inadequate practice of colonoscopy is always an alarming problem in lower-income countries. In developed countries, colonoscopy behaves better in service accessibility and quality (7–10), which largely benefits from their adequate resource and rigorous quality improvement (11, 12). But in developing countries, the capacity of the colonoscopy and its role in screening for CRC is still prominently limited, such as Brazil (13), Indonesia (14), and some African countries (15–17). The insufficient capacity to perform colonoscopy including infrastructure, utilization, and quality could be adverse to clinical practice and limit the efficacy of endoscopic screening for the reduction of the CRC burden (18). It is important to supervise colonoscopy practice using a national survey. Nonetheless, there is an absence of reliable nationwide data in China, especially the analysis of specific factors affecting its practice.

For the present study, we comprehensively reported and assessed the overall development of colonoscopy based on two national censuses in China. Moreover, we identify various factors associated with the regional variations and quality of colonoscopy. Our findings will provide important references for policymakers to identify areas that need strengthening, and then to effectively regulate population-based colonoscopy service strategies in the future.

## Method

### Organization

The Chinese Digestive Endoscopy Census was conducted twice in 2013 and 2020, and the results were incorporated into the National Report on the Services, Quality, and Safety in Medical Care System. The details of the census in 2013 and some of its results have been discussed previously (19, 20). The census in 2020 was launched and conducted by National Digestive Endoscopy Improvement System (NDEIS) between September 2020 and November 2021, which was supervised by the National Health Commission (NHC) of the People’s Republic of China. A census expert panel consisting of experts from the NDEIS, NHC, and Endoscopist Branch of the Chinese Medical Doctor Association was responsible for the routine work. After a pilot survey in Shanghai, all hospitals that performed digestive endoscopy in China were considered subjects and invited to complete a predesigned structured questionnaire online according to the retrospective data in 2019, which was facilitated by local health authorities and provincial Digestive Endoscopy Quality Control Centers. The study protocol was approved by the NHC of China and all included hospitals provided consent.

### Data collection and verification

To complete the questionnaire, every hospital designated personnel to log into a unique account online (https://www.ndeis.cn/). The accuracy of the submitted data was automatically assessed by logical tests using computer programs. First, when entering data in the questionnaire, the system automatically prompts for further confirmation of missed data and incorrectly filled outliers based on a simple algorithm. Then, the accuracy of the data was assessed by a rigorous and sophisticated logical test. The program was rigorously pre-designed by digestive endoscopy experts in cooperation with computer experts, which was based on the practical consensus of endoscopy disciplines. In Chinese practices, there is a strong correlation between the volume of endoscopy and basic information of hospitals, such as the number of endoscopists, number of endoscopic operating rooms, number of endoscopic devices, scale of gastroenterology department and hospital. This program can automatically retrieve unconventional data and send it back to the corresponding hospital and quality control center for confirmation when required. For example, a primary hospital with a small number of endoscopists and endoscopic devices submitted data on volume beyond its capacity.

Furthermore, ten percent of the hospitals were randomly selected by each provincial Digestive Endoscopy Quality Control Centers, in which endoscopic reports were drawn and checked for manual validation. As a result, the questionnaire was returned to the hospital and redone until to pass all the verifications, if the data were incomplete or failed to pass any verification. Finally, all the data were collected and analyzed in NDEIS, Shanghai.

### Real world information

Nationwide, there were total of 6,128 and 7,740 hospitals performed digestive endoscopy in mainland China. All colonoscopy-related information in this study came from these results, including information on hospitals, endoscopists, device, volume, and quality indicators. We further performed an association analysis between CRC incidence and colonoscopy practice based on the data of national cancer registry and Chinese Digestive Endoscopy Census in 2019. Data of new cases and deaths of CRC in a total of 487 cancer registry regions were extracted from the China Cancer Registry Annual Report in 2019 (21), which covers 381,565,422 inhabitants in China.

To identify the other potentially relevant factors affecting the practice of colonoscopy, various socioeconomic data were collected from multiple sources. The socio-demographic indexes (SDI) of 31 provincial regions were derived from Global Burden of Disease Results and were used to rank in the slope index of inequality (22). National and provincial populations in 2012 and 2019 were from National Bureau of Statistics and were used to calculate the utilization rate of colonoscopy (23). The gross domestic product (GDP) per capita and population in regions of cancer registries were collected from the open statistical data and financial statements.

### Statements and definitions

The two national censuses were mainly conducted in mainland China, covering 31 provincial regions (provinces, autonomous areas, and municipalities), including Anhui, Beijing, Fujian, Gansu, Guangdong, Guangxi, Guizhou, Hainan, Hebei, Henan, Heilongjiang, Hubei, Hunan, Jilin, Jiangsu, Jiangxi, Liaoning, Inner Mongolia, Ningxia, Qinghai, Shandong, Shanxi, Shaanxi, Shanghai, Sichuan, Tianjin, Tibet, Xinjiang, Yunnan, Zhejiang, and Chongqing. In China, hospitals are classified into tertiary, secondary, primary, and ungraded ones according to their scale and medical quality (24). Hospitals also can be divided into specialized or general hospitals and state-owned and private hospitals. Currently, the higher education background of physicians consists of four parts in China, specialized, bachelor’s, master’s, and doctoral degree.

In this study, volume refers to the total cases that underwent colonoscopy for all indications of screening, surveillance, diagnosis, and treatment. The utilization rate was defined as the total volume per 100,000 inhabitants in a certain year, which was used as the main index to assess the adequacy of colonoscopy utilization. The adenoma detection rate (ADR) was loosely defined as the unadjusted percentage of patients who have one or more precancerous polyps (adenomas) detected during all colonoscopies. The use of this kind of overall ADR rather than screening-only ADR could simplify measurement and increase the number of examinations available to measure ADR (25). In this study, the ADR was regarded as the key metric of colonoscopy quality and the representative indicator to analyze the influencing factors.

### Statistical analysis

Student’s t-test was used to identify the difference between the data of cancer registry regions and the whole nation to show the representativeness of our analysis. Non-constant variance score test was performed to determine the heteroscedasticity and the suitability for the slope index. Nonparametric correlation statistical tests (two-sided Spearman’s test) were performed to analyze the correlations between potential factors and regional utilization rates. A multiple logistic regression model was established based on the parameter from the statistically significant results of Spearman’s test. The value of ADR was included in a mixed linear model with potentially relevant parameters to estimate the factors influencing colonoscopy quality. Statistical analysis was performed using SPSS version 27.0 for Windows (IBM Corp., Armonk, NY, USA) and R version 4.2.2. In this study, statistical significance was set at a two-sided P value < 0.05.

## Results

### Hospitals, endoscopists, and devices for colonoscopy

From 2012 to 2019, the number of hospitals performing colonoscopy increased from 3,210 to 6,325 (1.97-fold). The number of hospitals per 100,000 inhabitants increased from 0.24 to 0.45. Specifically, 61.2% and 55.6% of them were secondary hospitals in 2012 and 2019, respectively, and 38.1% and 34.5% of them were tertiary hospitals. Primary and ungraded hospitals remained the lowest contribution but experienced the highest growth from 0.72% to 9.9%.

Nationwide, there were 26,203 and 39,638 digestive endoscopists respectively in 2012 and 2019 (1.51-fold). The majority of them were concentrated in tertiary hospitals (63.3% and 57.6%). The number of endoscopists per 100,000 inhabitants increased from 1.9 to 2.8 (1.47-fold). Regarding educational background, the percentage of endoscopists with master’s and doctoral degrees increased from 29.7% to 36.2%. The detailed characteristics of hospitals, endoscopists, and devices are shown in **Table 1**.

**Table 1:** The characteristics of hospitals, endoscopists, and devices of colonoscopy in China.

	2012	2019	P for difference
Hospital grading			<0.001
Tertiary hospitals	1223	2180	
Secondary hospitals	1964	3518	
Other hospitals*	23	627	
Hospital ownership			0.005
State-owned hospitals	2832	5449	
Private hospitals	378	876	
Hospital category			<0.001
General hospital	3144	5939	
Specialized hospital	66	386	
Educational background of endoscopists			<0.001
Master’s or doctoral degrees	7790	14350	
Bachelor’s degree or below#	18413	25288	
Devices			<0.001
Digestive endoscopy processor	12472	17374	
Colonoscope	9129	24725	

* Other hospitals including primary and ungraded hospitals. # Such as junior college or technical secondary schools.

### Colonoscopy volume

Overall, the volume of colonoscopy in mainland China increased from 5.83 to 12.92 million cases (2.21-fold), and the median volume per hospital changed from 811.5 (IQR, 300.0-2054.3) to 800.0 (IQR, 300.0-2181.0) cases. Specifically, 76.3% and 74.4% were performed by tertiary hospitals, 23.1% and 22.6% were performed by secondary hospitals, and only 0.5% and 3.0% were finished by primary and ungraded hospitals in 2012 and 2019, respectively. Meanwhile, the volume of colorectal endoscopic submucosal dissection increased from 31.4 to 114.4 thousand cases (3.64-fold).

### Utilization rate and inequality of colonoscopy service

**Figure 1:**
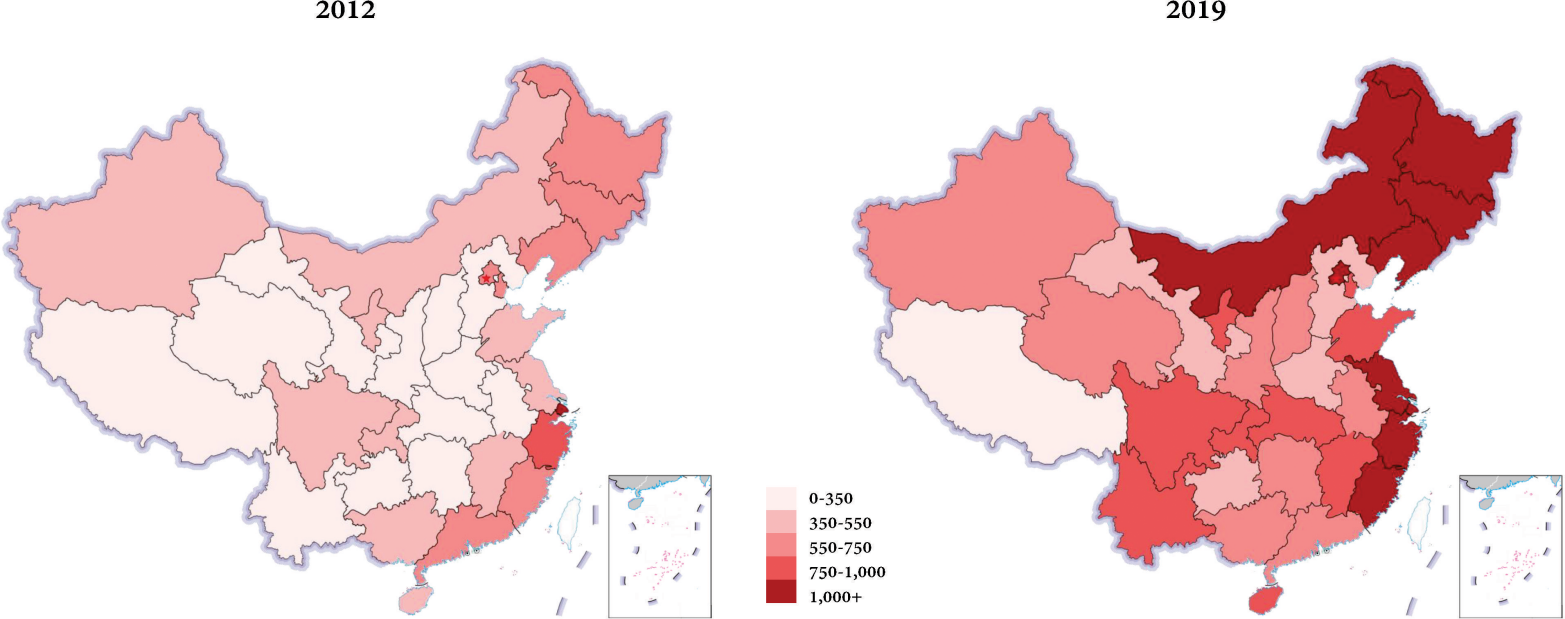
Regional distribution of the utilization rates of colonoscopy in mainland China in 2012 and 2019. Nationwide, the adequacy of the colonoscopy utilization had been improved apparently, but the regional rates varied widely both for the period 2012 and 2019.

**Figure 2:**
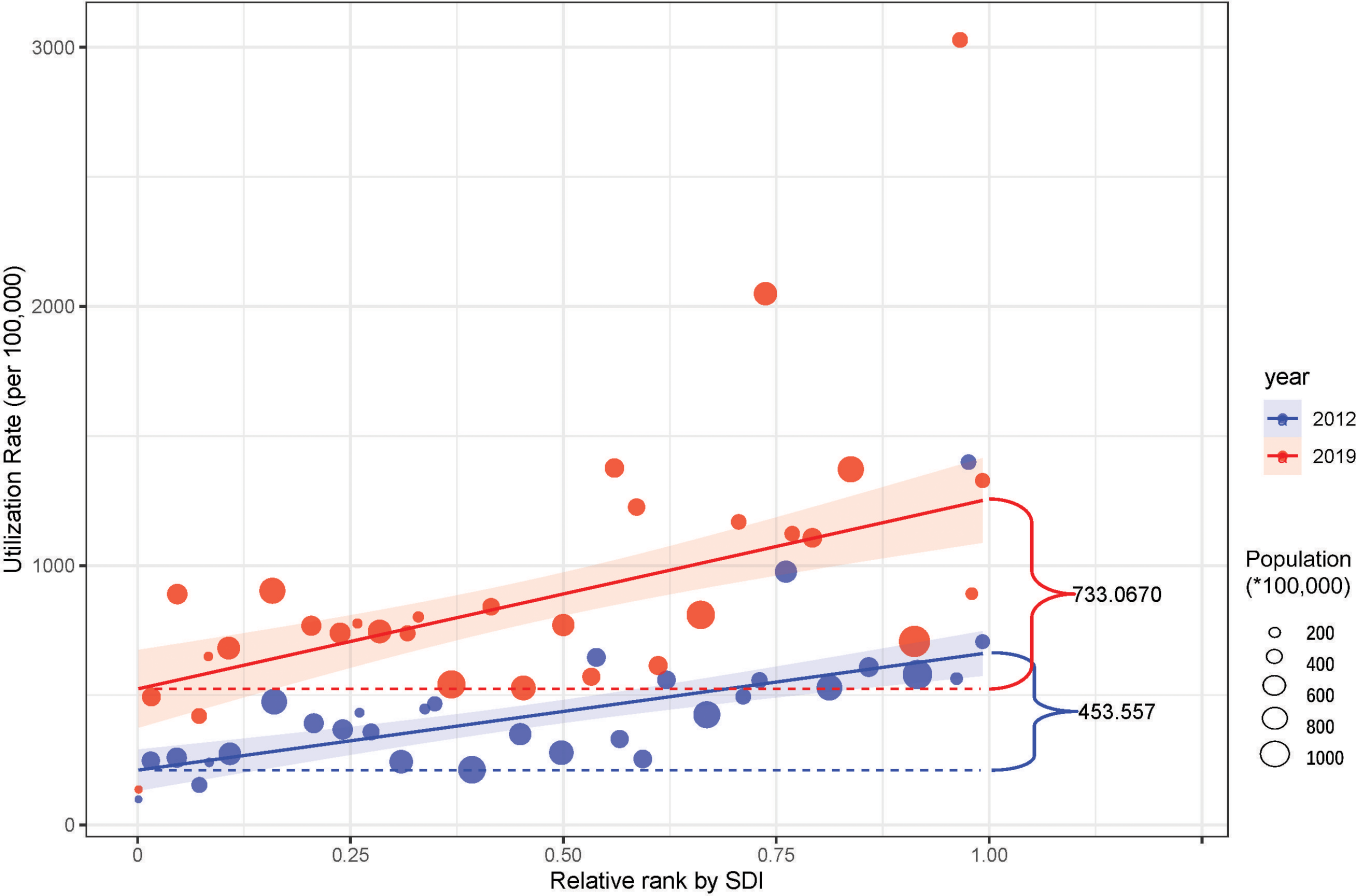
The slope index of inequality in regional colonoscopy service in China. The utilization rate was used to represent the availability of the colonoscopy and the regions were ranked by SDI. The indexes imply that the highly developed regions not only have a better basis but also the faster improvement of colonoscopy, which led to the aggravation of the inequality.

The national utilization rate rose from 436.0 to 914.8 per 100,000 inhabitants (2.10-fold). Meanwhile, the utilization rates in 31 provincial regions also experienced growth respectively. (**Figure 1**) However, the regional rates varied widely, which revealed significant inequalities in colonoscopy services. Over the past seven years, these inequalities were being extended rather than narrowed. (**Figure 2**)

In 2019, 2,631 hospitals were performing digestive endoscopy located in regions of 487 cancer registries and 2,277 (86.55%) of them provided colonoscopy service. The average utilization rate among these regions showed no significant difference from the national rate (910.5 vs 914.8, p=0.933). The overall ADR of these regions was also not significantly different from the national ADR (18.2% vs 18.1%, P=0.682).

Significant bivariate correlations were observed between the utilization rates in different regions and the number of endoscopists per 100,000 inhabitants (ρ=0.783, P<0.001), the number of tertiary hospitals performing colonoscopy per 100,000 inhabitants (ρ=0.679, P<0.001), the percentage of endoscopists with master’s and doctoral degrees (ρ=0.603, P<0.001), GDP per capita (ρ=0.517, P<0.001), the incidence of CRC (ρ=0.513, P<0.001), the number of hospitals performing colonoscopy per 100,000 inhabitants (ρ=0.348, P<0.001).

To assess potential factors affecting the regional utilization rates, multiple logistic regression was performed with parameters derived from the above statistically significant results (**Table 2**). As a result, endoscopists per 100,000 inhabitants (odds ratio [OR] 2.76, 95%CI 1.92-3.95; P<0.001), tertiary hospitals performing colonoscopy per million inhabitants (OR 1.80, 95%CI 1.45-2.24; P<0.001), incidence of CRC/100,000 inhabitants (OR 1.08, 95%CI 1.04-1.12; P<0.001), GDP per capita/10,000 RMB (OR 1.24, 95%CI 1.11-1.39; P<0.001) were observed to positively associate with more adequate colonoscopy. Additionally, the percentage of endoscopists with master’s and doctoral degrees and the total number of hospitals performing colonoscopy per million inhabitants were not significant in the final model (P>0.05).

**Table 2:** Multiple logistic regression analysis of factors associated with the regional utilization rate.

Variable	OR	95% CI	P
GDP per capita per 10,000 RMB	1.244	1.114-1.389	<0.001
Incidence of CRC per 100,000 inhabitants	1.081	1.044-1.119	<0.001
Hospitals per million inhabitants	0.954	0.827-1.100	0.517
Tertiary hospitals per million inhabitants	1.801	1.446-2.243	<0.001
Endoscopists per 100,000 inhabitants	2.755	1.920-3.953	<0.001
High percentage of endoscopists with master’s and doctoral degrees	1.100	0.443-2.730	0.838

The utilization rates were regarded as dichotomous variable based on the national average.

### Colonoscopy quality and factors affecting ADR

Nationwide, the quality of colonoscopy experienced remarkable growth. The cecal intubation rate increased from 83.9% in 2012 to 94.4% in 2019, and the ADR increased from 16.3% to 18.1%. The quality results of hospitals with different grades are demonstrated in **Table 3**.

**Table 3:** The colonoscopy quality of hospitals with different grades in China.

Hospital grading	Cecal intubation rate, %		ADR, %	
	2012	2019	2012	2019
Tertiary hospitals	86.3	95.4	16.9	19.1
Secondary hospitals	76.1	89.5	14.2	16.1
Other hospitals*	67.7	88.3	13.9	15.5
Total	83.9	94.4	16.3	18.1

* Other hospitals including primary and ungraded hospitals.

**Figure 3:**
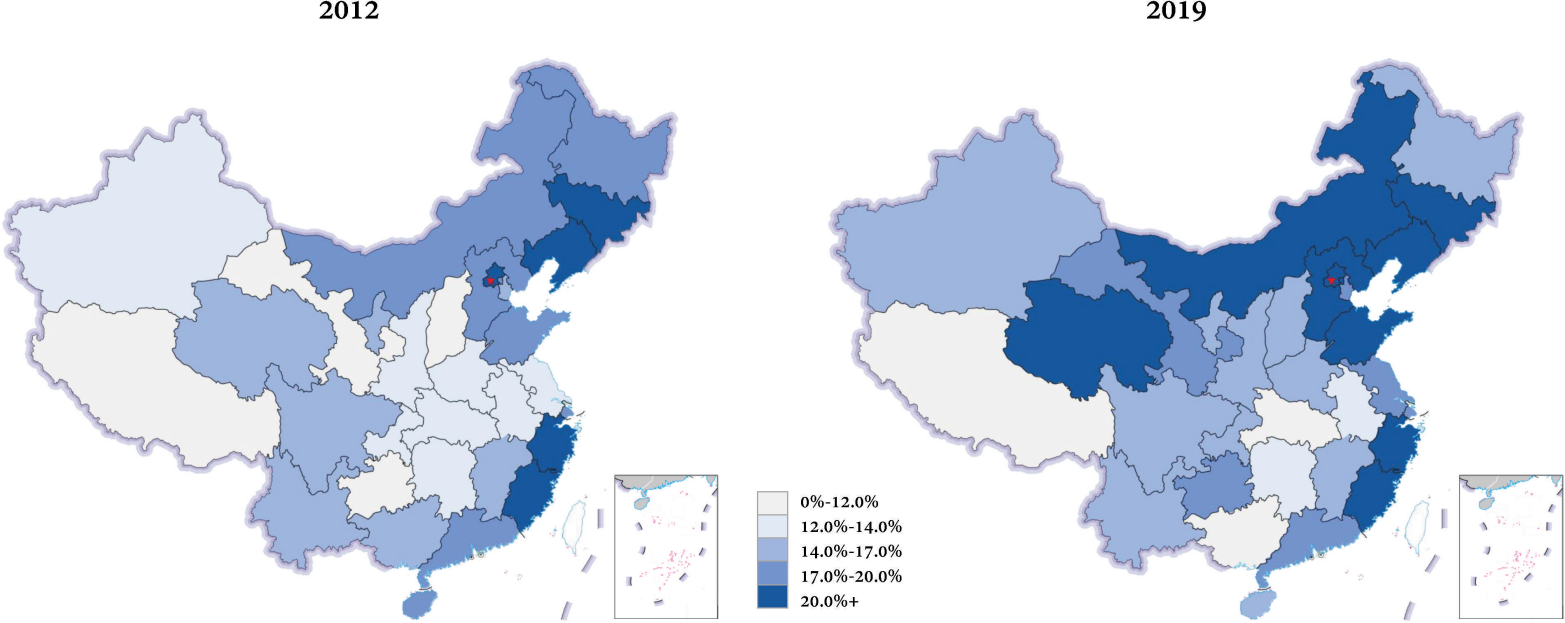
Regional distribution of the ADR of colonoscopy in mainland China in 2012 and 2019. The overall level of ADR had been risen, but the regional disparity was still substantial.

The ADR showed a substantial disparity in regional distributions. (**Figure 3**) In the multivariate mixed model, the hospital grading (tertiary, β=0.0837; secondary, β=0.0759, ungraded, β=0.0318), the percentage of endoscopists with master’s and doctoral degrees (β=0.0041), GDP per capita (β=0.0013), and volume of colonoscopy showed significantly positive correlations to the ADR in different hospitals (P<0.05). However, the number of endoscopists (P=0.794) and incidence of CRC (P=0.469) showed no relevance to the colonoscopy quality (**Table 4**).

**Table 4:** Mixed linear model of factors associated with ADR.

Parameters	β	95% CI	P
Intercept	0.04214	0.02254-0.06173	<0.001
Tertiary hospital	0.08368	0.06261-0.10476	<0.001
Secondary hospital	0.07585	0.05740-0.09430	<0.001
Ungraded hospital	0.03179	0.00584-0.05774	0.016
Number of endoscopists	0.00015	-0.00087-0.00127	0.794
Percentage of endoscopists with master’s and doctoral degrees (per 10%)	0.00412	0.00185-0.00638	<0.001
GDP per capita (per 10,000 RMB)	0.00125	0.00028-0.00222	0.011
Incidence of CRC (per 1/100,000)	0.00009	-0.00016-0.00035	0.469
Volume of colonoscopy (per 100 cases)	0.00023	0.00036-0.00043	0.021

The parameters of primary hospitals are redundant in the mixed linear model.

## Discussion

This is the first and largest study that presented the national development and status quo of colonoscopy in China. Nationwide, colorectal cancer is becoming a huge public health problem. Undoubtedly, colonoscopy is the most effective measure in terms of diagnosis and removal of early cancer and precancerous lesion with relatively low invasiveness. In this article, we identified the huge progress of colonoscopy over the past years in China, which mainly benefited from the rapid economic development and the promotion of health policy. Meanwhile, the potential factors affecting the practice of colonoscopy were assessed based on various real word information. Our findings could identify specific situations where the problem is and could provide important references to better the quality improvement and yield of screening. Furthermore, it provides a convincing reference for international researchers.

Despite the inspiring development, the following aspects should be considered further in China. First, inequality of colonoscopy services. The utilization rate was paled in comparison with the estimated rate in some developed countries of the corresponding period (4334.6 in the USA (12), 4074.8 in Korea (8), and 2725.4 in Japan (24)), which meant it did not meet the huge CRC screening needs in China. In a previous study conducted in an advanced digestive endoscopy center of a tertiary hospital, the mean number needed to screen for all types of adenomas and CRCs in an average-risk Chinese population were 6.9 and 169.5, respectively (26). In a sense, the total colonoscopy volume in China, even with the advanced quality, seemed to fail to meet the screening needs. Besides, it also showed a significant disparity in regional distributions. Although various factors were positively relevant, we found that a higher utilization rate was observed for regions with more tertiary hospitals, more endoscopists, higher GDP per capita, and higher incidence of CRC. In other words, our overall unsatisfactory screening of colorectal cancer was largely attributed to insufficient and inequal colonoscopy resources and services. Policymakers can promote the accessibility of local endoscopy services by improving those modifiable factors so that regional colonoscopy capability can be sufficient for screening and treatment of CRC. These challenges can be gradually mitigated through the identification of the issues and concerted efforts from governments and healthcare institutions.

Second, the overall quality of colonoscopy was imbalanced. The cecal intubation rate grew from 75.8% to 94.4%, which was catch up with or even surpass some developed countries, such as the UK (92.3%), Netherlands (92.4%), and Italy (83.0%) (7, 27, 28), and developing countries, such as Brazil (94.0%) (13). However, the huge gap of ADR is still visible. The ADR was 38.1% in the United States (11), 28.7% in Japan (10), 32.1% in UK (7), 39.1% in Italy (9), and 36.6% in Brazil (13). Next, measures should be taken to improve adenoma detection, such as the deployment of a registry system collecting uniform data for measurements of endoscopy quality index (29, 30), effective bowel preparation, and incorporation of artificial intelligence and novel technique (31, 32). Besides, domestic results varied widely. Our analysis implied that hospital grading, educational background of endoscopists, economic level, and volume were key factors for improving ADR. What exceeded our expectations was that the incidence of CRC had no impact on the detection of adenoma, and expertise rather than the number of endoscopists was necessary to improve the ADR. Those findings were consistent with the conclusions suggested by previous studies. In a retrospective research conducted in the United States (33), the ADR showed significant difference among individual endoscopists but no significant differences between all colonoscopies and screening colonoscopies with different incidence.

Third, more attention should be paid to endoscopists training. By analysis, we identified the unique role of endoscopists in colonoscopy practice. Their quantity partly determines the colonoscopy utilization, and their educational backgrounds were associated with the quality. Moreover, endoscopist-related characteristics that influence the quality of colonoscopy have been reported (34). More widespread training and education programs for practicing endoscopists should be launched to improve their professional competence for meeting the increasing demand.

Fourth, the capacity of primary healthcare requires facilitation. Those secondary and primary hospitals were found to have the advantage of quantity and availability. Primary care hospitals are necessary to play a greater role to remedy the deficiency in cancer early detection and early treatment. Additionally, the diagnostic yield was not optimal using colonoscopy screening in high-risk populations given the relatively low participation rate in China (35). Reasonable national allocation of colonoscopy resources provides more convenient and accessible services for the general population.

This study has several limitations. First, recall bias cannot be avoided because of the retrospective design of the census. Second, only the overall volume of colonoscopy was collected, rather than clearly classified for screening, surveillance, and treatment purposes. Third, other real information in the analyses were from domestic open access rather than the census itself. Finally, the utilization rates of other countries were estimated from studies and reports with different designs and coverage. Therefore, the comparison just drew an outline.

In conclusion, a better understanding of national practice of colonoscopy may be the first step toward successful implementation of quality improvement. Our results will provide important references for designing effective nation-based colonoscopy strategies. At present, opportunities and challenges are facing Chinese colonoscopy simultaneously, which makes policy- and hospital-level actions imminently.

## Data availability statement

The raw data supporting the conclusions of this article will be made available by the authors, without undue reservation.

## Author contributions

Y-FJ: Conceptualization, Data curation, Formal Analysis, Investigation, Methodology, Project administration, Software, Supervision, Validation, Visualization, Writing – original draft, Writing – review & editing. Z-YC: Conceptualization, Data curation, Formal Analysis, Investigation, Software, Visualization, Writing – review & editing, Methodology, Writing – original draft. YG: Conceptualization, Formal Analysis, Investigation, Methodology, Resources, Software, Writing – review & editing, Writing – original draft. C-TY: Conceptualization, Investigation, Methodology, Software, Writing – review & editing. H-SJ: Conceptualization, Investigation, Methodology, Writing – review & editing. T-LW: Conceptualization, Investigation, Methodology, Software, Writing – review & editing. YD: Conceptualization, Investigation, Methodology, Writing – review & editing. HL: Conceptualization, Investigation, Methodology, Project administration, Supervision, Writing – review & editing. T-JW: Conceptualization, Investigation, Methodology, Project administration, Resources, Software, Supervision, Writing – review & editing. WW: Conceptualization, Investigation, Methodology, Project administration, Software, Supervision, Writing – review & editing. RW: Investigation, Resources, Writing – review & editing. Z-SL: Conceptualization, Funding acquisition, Investigation, Methodology, Project administration, Resources, Supervision, Writing – review & editing. LX: Data curation, Formal Analysis, Investigation, Methodology, Project administration, Resources, Supervision, Writing – original draft, Writing – review & editing. L-WW: Conceptualization, Formal Analysis, Funding acquisition, Investigation, Methodology, Project administration, Resources, Supervision, Visualization, Writing – review & editing, Writing – original draft.
